# A meta-analytic review of Mandarin tone perception: tone height and tone contour, prosodic background, and L2 experience

**DOI:** 10.3389/fpsyg.2025.1670858

**Published:** 2026-03-10

**Authors:** Xin Cui, Hong Zhao

**Affiliations:** Department of English, School of Foreign Languages, East China University of Science and Technology, Shanghai, China

**Keywords:** L2 experience, meta-analysis, prosodic background, tonal features, tone perception

## Abstract

This meta-analysis synthesizes data from 18 empirical studies to examine how tonal features of height and contour, native prosodic background, and second language (L2) experience affect the perception of Mandarin lexical tones. Results revealed overall large effect sizes, indicating that variability in tone perception is statistically robust and theoretically significant. Importantly, substantial heterogeneity across studies was explained by key methodological moderators, including task type, stimulus type, outcome measure, sample size, and gender composition. Studies using passive hearing tasks, synthesized stimuli, accuracy-based outcome measures and more female participants consistently produced larger effect sizes, suggesting enhanced perceptual salience and reduced processing demands. Moreover, moderate sample sizes were sufficient to detect meaningful effects, particularly in cross-linguistic comparisons. Analyses confirmed three major findings: (1) Tone height and pitch span critically shaped perceptual accuracy, with high-pitched tones and those with greater pitch span perceived more accurately; (2) Native prosodic background significantly modulated tone perception, with pitch-accent and tone language speakers generally outperforming non-tonal listeners. However, perceptual assimilation could either facilitate or interfere with tone perception in tone language listeners when similar categories existed in their L1 and the target language; (3) L2 experience was a positive predictor of tone perception accuracy; however, improvements followed a nonlinear, tone-specific trajectory. Tone 2 and Tone 3 remained persistently challenging to differentiate, with substantial progress typically observed only at advanced proficiency levels. Together, these results underscore the complex interplay between acoustic properties, language background, and learning experience in Mandarin tone perception, offering valuable insights for both theoretical modeling and pedagogical practice in second language acquisition of tones.

## 1 Introduction

Successful verbal communication depends critically on the listener’s ability to accurately extract and interpret a rich array of acoustic cues embedded within the speech signal. These cues encompass not only segmental information, such as consonants and vowels, but also suprasegmental features that operate to convey prosodic, emotional, and lexical distinctions. Among these suprasegmental features, lexical tone plays a particularly salient role in tonal languages, where pitch variations across syllables serve to differentiate word meanings. A classic example is found in Mandarin Chinese, where the syllable “ma” can mean “mother” (Tone 1), “hemp” (Tone 2), “horse” (Tone 3), or “to scold” (Tone 4), depending solely on its tonal contour (Zhang, 2014). This widespread use of tone to distinguish lexical meaning is far from an isolated phenomenon; indeed, it is estimated that over 70% of the world’s languages employ some form of tonal contrast to signal differences in meaning (Yip, 2002; Maddieson, 2013).

Given the broad prevalence of lexical tone and its critical communicative function, accurate perception of tonal distinctions is essential for effective daily communication. This importance has, in turn, triggered extensive research interest into the mechanisms underlying tone perception. Although tone perception is influenced by a wide range of linguistic factors—including lexical context, tone sandhi, segment–tone interactions, and dialectal variation (Pelzl, 2019)—the current review focuses on three perceptual dimensions that are both theoretically central and empirically tractable for meta-analysis: (1) tonal features represented by tone height and tone contour, (2) native prosodic background, and (3) L2 experience. These dimensions have been the most consistently examined across experimental studies, provide sufficient effect-size statistics for quantitative synthesis, and are directly relevant to identifying potential cross-linguistic universals in tone perception.

### 1.1 The potential influence of tone height and tone contour on tone perception

The first key question concerns whether different tonal features lead to varying levels of perceptual difficulty. Based on pitch dynamics, *tone height* (the register or pitch range a tone occupies) and *tone contour* (the trajectory or slope of pitch change) have been identified as two primary tonal features in tone perception (Hyman, 2011; Gandour, 1983; Khouw and Ciocca, 2007; Wong and Diehl, 2003; Yip, 2002). Accordingly, two important questions arise based on the two features: what roles do height and contour play in tone perception, and how are they integrated during perceptual processing?

Previous research has largely addressed height and contour separately, often focusing on specific language contexts. For example, from a comparative perspective, speakers of tone languages with rich level tone inventories, such as Cantonese, have been found to rely more on tone height (Burnham et al., 2022; Wong and Diehl, 2003); while speakers of languages dominated by contour tones, such as Thai and Vietnamese, tend to attend more to tone contour (Khouw and Ciocca, 2007; Gandour, 1983). While these studies provide valuable insights, they typically examine one factor at a time and do not offer an integrated account of how both tone height and contour jointly shape tone perception.

One reason for this limitation may lie in the complexity of the tone systems studied. Languages like Cantonese contain six to nine lexical tones, many of which occupy narrow and overlapping acoustic spaces (Gandour, 1983). This overcrowding can lead to confounding perceptual effects, making it difficult to disentangle the relative contributions of pitch height and contour, and to generalize findings across languages.

To mitigate these confounding factors and better reveal the general roles of tone height and contour in tone perception, it is advantageous to examine a tone system that has relatively few tones, and features well-distributed acoustic spacing. Ideally, this system should also allow for cross-linguistic comparison by including listeners from diverse native language backgrounds. Mandarin Chinese offers such an ideal case. With only four lexical tones—Mandarin provides a balanced yet typologically informative system for exploring tone perception. The tonal inventory spans a range of pitch patterns with minimal acoustic overlap, and the language is widely studied among second-language learners from various prosodic backgrounds, including stress, pitch-accent, and tone languages (Liu, 2020).

Therefore, the current study focuses on Mandarin tone perception to conduct a meta-analytic synthesis that can clarify the respective and potentially interactive influences of tone height and contour. By drawing on data from diverse populations and experimental paradigms, this analysis aims to offer a more integrated understanding of how tonal features of height and contour shape tone perception across language users.

### 1.2 Conflicting evidence on the influence of native prosodic background

A second line of inquiry concerns how listeners’ native prosodic background modulates their ability to perceive non-native tones. From a typological perspective of prosody, human languages can be broadly categorized into stress, pitch-accent, and tone languages based on how they utilize pitch in the prosodic system (Gussenhoven, 2004). Accordingly, the second question asks whether speakers of the three types differ systematically in their perception of lexical tones based on their native prosodic background.

Some researchers argue that these typological differences give rise to perceptual asymmetries, whereby speakers of tone and pitch-accent languages demonstrate superior tone perception compared to speakers of non-tonal languages. This view is supported by a substantial body of research (Bent et al., 2006; Burnham et al., 2015; Chang et al., 2017). Neural evidence further reinforces this claim, suggesting that tone language speakers may engage pitch-sensitive cortical regions more efficiently than speakers of stress-based languages during tone perception tasks (Liang and Du, 2018).

However, this perspective has been challenged by findings that reveal inconsistent differences among listeners from the three typological backgrounds. For example, Gao (2024) found no significant advantage for Swedish (pitch-accent) learners over Danish (non-tonal) learners in identifying Mandarin tones after equivalent exposure. Similarly, Cantonese (tonal) listeners performed worse than English (non-tonal) listeners in distinguishing between Mandarin Tone 2 and Tone 3 (So and Best, 2010).

Taken together, these conflicting results suggest that while prosodic typology provides a useful starting point, there is still no clear consensus regarding the precise role of L1 prosodic background in shaping non-native tone perception—particularly with respect to the perception of Mandarin lexical tones.

### 1.3 Inconsistent findings on the role of L2 experience in tone perception

A third major question in the field concerns how second language (L2) experience shapes the perception of lexical tones. A growing body of research has demonstrated that exposure to a tone language can significantly improve tone perception abilities in adult L2 learners (Wang et al., 2003; Pelzl et al., 2019). However, the nature and trajectory of this learning process remain under debate. One unresolved issue is whether tone perception improves in a linear fashion with increasing exposure or whether it follows a nonlinear trajectory, possibly characterized by plateaus or periods of stagnation, similar to patterns observed in segmental phoneme learning (Best and Tyler, 2007; Flege and Bohn, 2021). Well-established findings from segmental phonology research—such as the effects of perceptual assimilation (Best, 1995), input variability (Logan et al., 1991), and categorical perception (Peng et al., 2010)—have begun to be tested in tonal domains, yet results have been inconclusive or inconsistent. For example, while L1 prosodic background seems to predict initial performance, it is less clear whether it constrains long-term learning outcomes (So and Best, 2010). Similarly, variability in tonal input (e.g., multiple talkers, natural speech) appears to enhance learning in some cases (Silpachai, 2020), but not others (Zhang et al., 2018).

Taken together, these conflicting results highlight the complexity of tone learning in a second language and suggest that current models of L2 phonological acquisition may not fully capture the dynamics of tone perception development. A systematic synthesis of existing empirical findings is therefore urgently needed to clarify whether L2 tone learning follows identifiable patterns—particularly, how many years of experience are required to achieve significant perceptual sensitivity, and whether L2 experience differentially affects the perception of various tone types.

### 1.4 The current study: toward a meta-analytic understanding of tone perception through Mandarin

As reviewed above, tone height, tone contour, native prosodic background, and second language experience all appear to play crucial roles in lexical tone perception. Yet, despite growing empirical attention, considerable divergence remains in how these factors operate. Specifically, there is still no consensus on whether distinct tone height and tone contour engage shared or specialized mechanisms, whether learners’ first language prosodic backgrounds reliably predict non-native tone perception outcomes, and how L2 experience modulates perceptual development over time. These unresolved issues are further complicated by the potential interactions among the three dimensions, making it challenging to derive a unified understanding of tone perception across populations and learning contexts.

A promising strategy to address these gaps is to focus on the perception of lexical tones in a single tone language that offers both typological richness and broad learner accessibility. Mandarin Chinese is particularly well suited for this purpose. It possesses four distinct tones: a high-level tone (Tone 1, T1), a rising tone (Tone 2, T2), a falling tone (Tone 4, T4), and a more acoustically complex dipping tone (Tone 3, T3). This tonal inventory spans a spectrum of pitch complexity and combines both height and contour features, making it an ideal testbed for exploring differences in perceptual processing. Additionally, Mandarin is one of the most widely studied second languages globally, with learners from diverse L1 prosodic backgrounds—including stress, pitch-accent, and other tone languages (Chang, 2011)—thus offering a robust basis for cross-linguistic comparison and analysis.

In this context, the present study aims to conduct a comprehensive systematic review and meta-analysis of empirical studies examining non-native perception of Mandarin tones. Compared with traditional narrative reviews that qualitatively summarize findings, meta-analytic methods offer a statistically rigorous approach to synthesizing results across studies, estimating overall effect sizes, and identifying potential moderators (Borenstein et al., 2009; Valentine et al., 2010). While earlier reviews on this topic have largely relied on descriptive summaries (e.g., Pelzl, 2019), only a few recent attempts have applied meta-analytic techniques. For example, Liang and Du (2018) employed meta-analysis to investigate the neural mechanisms underlying tone perception across different L1 backgrounds, demonstrating the effectiveness of meta-analytic methods in analyzing tone perception issues. However, no study has yet used this approach to thoroughly examine the effects of tonal features of height and contour, prosodic background, and L2 experience on tone perception. Therefore, this issue remains in urgent need of further supplementation and in-depth exploration.

To advance the field, the current study performs a targeted meta-analysis focusing exclusively on Mandarin tone perception by L2 learners from different prosodic backgrounds. The goal is to quantitatively evaluate: (1) whether tone height and tone contour, as two primary tonal features, affect perception accuracy differently across groups; (2) how native prosodic backgrounds systematically shape tone perception; and (3) whether and how L2 learning experience predicts perceptual outcomes. By aggregating and statistically modeling existing data, this study seeks to clarify longstanding debates, uncover consistent patterns across studies, and generate a more unified understanding of cross-linguistic tone perception.

## 2 Methods

### 2.1 Eligibility criteria

Studies were considered eligible if they were published in English, adopted an experimental method, and compared the perception of the four Mandarin lexical tones by participants with different first language (L1) backgrounds or varying levels of second language (L2) experience in Mandarin Chinese (with or without learning experience, or at different stages of L2 acquisition). The auditory stimuli had to consist of either naturally recorded Mandarin tones or synthesized tones based on real Mandarin tones. Identification and discrimination tasks were preferred, although passive auditory perception tasks were also acceptable. Eligible studies were required to be primary empirical research with original data, published in peer-reviewed journals, and contain sufficient statistical information to calculate effect sizes for comparisons involving tonal features of tone height and tone contour, L1 backgrounds, or L2 learning experience. Review articles, editorials, and previous meta-analyses were excluded. Neuroimaging and electrophysiological studies were included only if they reported adequate behavioral data related to tone perception.

### 2.2 Search strategy

A systematic search was conducted through five major electronic databases—Web of Science, PubMed, PsycINFO, PsycARTICLES, and ERIC—for studies published from January 2010 to June 2025. The search was limited to the last 15 years to ensure the relevance and methodological consistency of included studies, as research on L2 tone perception has expanded significantly and become more standardized during this period. We used combinations of the following keywords: “Mandarin tone” OR “Chinese tone,” AND “perception” OR “perceptual,” AND “second language” OR “L2” OR “foreign language.” We also manually screened the reference lists of all included studies and relevant review articles to identify additional studies.

### 2.3 Study selection and data extraction

**Figure 1:**
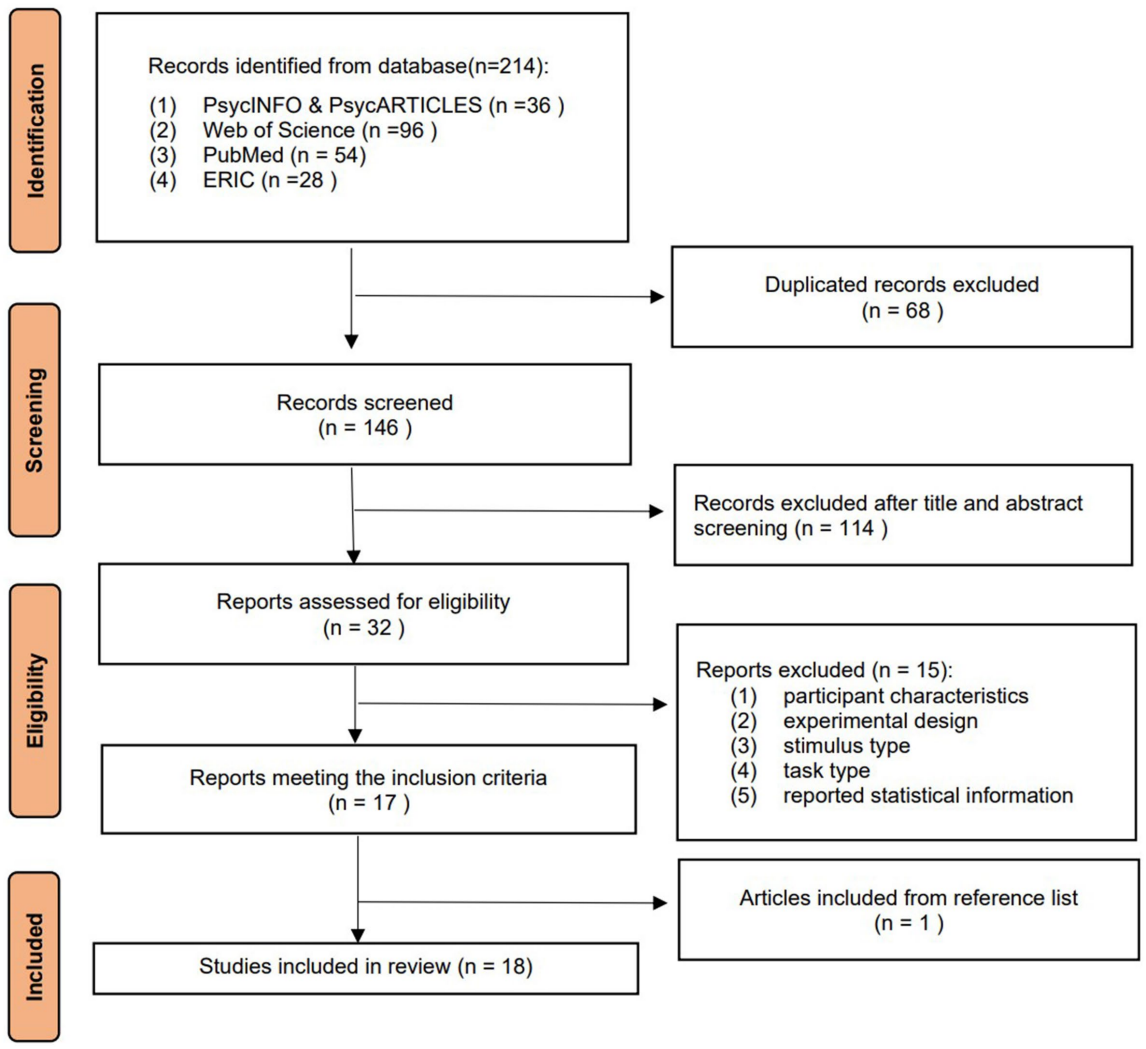
Procedure of different phases of the systematic review and meta-analysis.

As presented in Figure 1, the initial search yielded 214 records (Web of Science: 96; PubMed: 54; PsycINFO & PsycARTICLES: 36; ERIC: 28). After removing 68 duplicates, 146 records remained for title and abstract screening, from which 32 potentially eligible studies were identified. Full-text screening led to the exclusion of 15 studies—nine lacked extractable statistical information for computing effect sizes, and six employed tone-perception paradigms or stimuli that did not meet the criteria for the present review (e.g., studies based solely on synthetic tones with unrealistically wide pitch ranges, or perception tasks conducted under extreme noise conditions). A total of 17 studies were therefore retained, and one additional study was identified through reference-list search, resulting in 18 studies included in the final meta-analysis.

Among these 18 studies, some contributed data to one, two, or all three research questions in Section 1.4 in this review. Specifically, 12 studies provided analyzable effect sizes for tonal features (tone height and contour), 17 for native prosodic background, and 12 for L2 experience. Based on this distribution, the meta-analyses were conducted as three independent analyses, each performed on a dataset sufficiently large (n ≥ 10 studies) to yield reliable and interpretable effect-size estimates.

### 2.4 Statistical analysis

Meta-analyses were conducted in R (version 4.1.2) using in the *metafor* package (Viechtbauer, 2010). Effect sizes were expressed as Hedges’ *g*, with values of 0.2, 0.5, and 0.8 conventionally interpreted as small, medium, and large effects, respectively (Cohen, 2013; Lakens, 2013; Hedges, 1981). Effect sizes were computed using the ‘*escalc()*’ function in *metafor*, supplemented by manual calculations based on Hedges (1981) for studies in which effect sizes could not be directly derived from the reported statistics. Given the methodological and population variability across studies, a random-effects model was employed to estimate pooled effect sizes.

Between-study heterogeneity was assessed using Cochran’s *Q* and the *I^2^* statistic (Higgins and Thompson, 2002), computed by the *metafor* package (Viechtbauer, 2010). Significant *Q* values suggest non-random variability among study results, while *I^2^* quantifies the proportion of total variation due to true heterogeneity. Values of 25, 50, and 75% were interpreted as low, moderate, and high heterogeneity, respectively (Higgins et al., 2003).

Outliers were detected by identifying studies whose confidence intervals did not overlap with that of the pooled effect size (Harrer et al., 2019). Sensitivity analyses were conducted by removing these outliers and recalculating effect sizes. To evaluate study influence, a leave-one-out analysis was performed, in which each study was excluded in turn to assess its impact on the overall effect (Passos et al., 2015; Richardson, 2011; Lam et al., 2016).

Publication bias was first visually assessed using funnel plot asymmetry and then statistically examined with Egger’s regression test (Egger et al., 1997). When publication bias was indicated (*p* < 0.10), the trim-and-fill method was applied to adjust for potentially missing studies (Duval and Tweedie, 2000), and corrected effect sizes and 95% confidence intervals were reported.

Finally, meta-regression analyses were conducted using a mixed-effects model to explore the contribution of moderator variables to between-study heterogeneity in effect sizes. This study primarily examined seven potential moderators that may influence heterogeneity in effect sizes across studies: (1) Sample size, (2) Percentage of tonal L1 speakers, (3) Type of stimulus (e.g., real vs. synthetic tones), (4) Number of participant groups, (5) Number of tone tokens as stimuli, (6) Percentage of male participants, (7) Number of tone types (e.g., using two, three, or all four Mandarin tones). Moderators were included if sufficient data were available from at least four studies (Velikonja et al., 2019). All selected moderators correspond to the variables specified in the meta-regression models (i.e., variables specified after ‘effect_size ~’ in the model formulas, such as ‘effect_size ~ sample_size + stimulus_type + …’). The significance of the moderators was evaluated using *Q* statistics, *p*-values, and *R^2^* values to quantify the proportion of explained variance (Borenstein et al., 2009).

## 3 Results

To systematically examine how specific factors influence the perception of Mandarin lexical tones, three meta-analyses were conducted according to the research questions in 1.4: (1) The tonal features of height and contour involved in the experimental stimuli (including 12 studies), (2) the native language background of the participants (including 17 studies), and (3) their L2 learning experience (including 12 studies). Each meta-analysis was conducted using the procedures described in Section 2.4. Where significant heterogeneity in effect sizes was detected, follow-up moderator analyses were performed to explore potential sources of variability across studies.

### 3.1 Effects of tone height and tone contour

**Figure 2:**
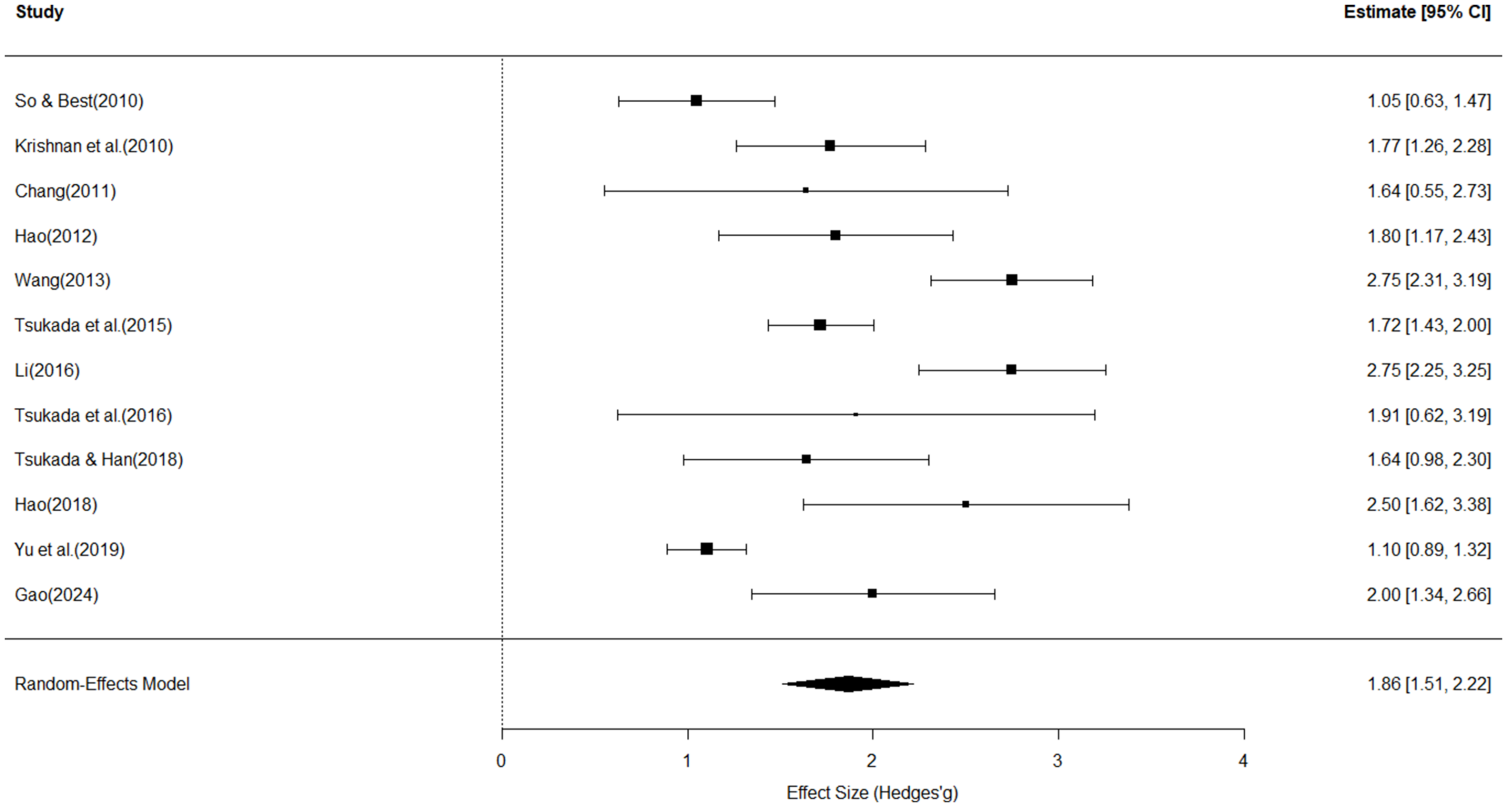
Forest plot for tone height and tone contour effects on perception, with individual and pooled effect sizes (Hedges’ *g*), standard errors and 95% confidence intervals (size of the rectangles represents study weight; horizontal line indicates 95% CI; diamond indicates pooled effect size; diamond width represents its CI).

Figure 2 summarizes the meta-analytic results examining the influence of tone height and tone contour on the perception of Mandarin lexical tones across 12 studies. The pooled effect size was large and statistically significant (effect size = 1.86, 95% *CI* [1.51, 2.22], *p* < 0.01; see Figure 2). The test for heterogeneity revealed significant variability in effect sizes among the included studies (*Q*(11) = 81.37, *p* < 0.01). The degree of heterogeneity was considerable, with an *I*^2^ value of 83.74%, indicating that over three quarters of the variance across studies can be attributed to true differences rather than sampling error. The estimated between-study variance was *τ*^2^ = 0.01 (*SE* = 0.0082), with a corresponding standard deviation of *τ* = 0.11, reflecting a moderate level of heterogeneity. Egger’s regression test was conducted to assess potential publication bias. The test did not reveal significant funnel plot asymmetry (*z* = 0.75, *p* = 0.45), suggesting that there was no statistically significant evidence of publication bias. The estimated effect size as the standard error approached zero was *b* = 1.58 (95% CI [0.776, 2.4]), indicating a stable and unbiased overall effect.

Subsequent moderator analyses showed that outcome measures significantly accounted for heterogeneity across studies (*Q_M_*(4) = 15.37, *R*^2^ = 51.28%, *p* = 0.004). Specifically, studies using accuracy rate as the outcome measure yielded significantly larger effect sizes compared to those using other outcome measures (*β* = 0.69, 95% CI [0.09, 1.30], *p* = 0.02).

In contrast, other moderators—including sample size, the percentage of tonal L1 speakers, the type of stimulus (e.g., real vs. synthetic words), the number of participant groups, the number of tone tokens, the percentage of male participants and the number of tone type—did not show significant effects on the magnitude of the effect sizes.

### 3.2 Effects of native prosodic background

**Figure 3:**
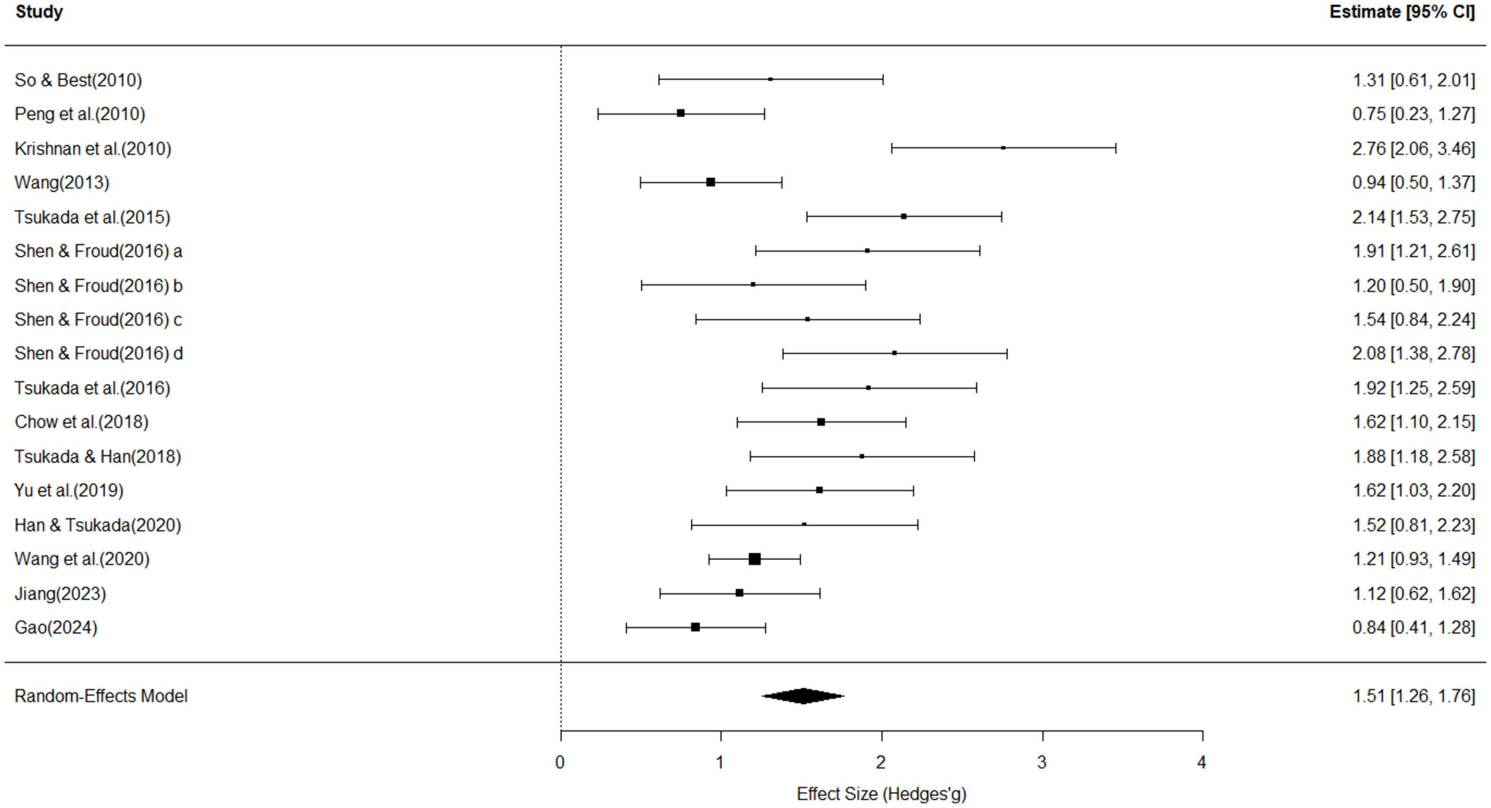
Forest plot for effects of prosodic background on perception, with individual and pooled effect sizes (Hedges’ *g*), standard errors and 95% confidence intervals (size of the rectangles represents study weight; horizontal line indicates 95% CI; diamond indicates pooled effect size; diamond width represents its CI).

Figure 3 presents the meta-analytic findings based on 17 studies examining the influence of participants’ native language background on their perception of Mandarin lexical tones. The overall effect size was statistically significant and of large magnitude (effect size = 1.51, 95% *CI* [1.26, 1.76], *p* < 0.0001; see Figure 3). The test for heterogeneity indicated substantial variation in effect sizes across studies (*Q*(16) = 51.59, *p* < 0.01). The *I*^2^ value was 70.79%, suggesting that over 70% of the total variance reflected true between-study differences rather than sampling error. The estimated between-study variance was *τ*^2^ = 0.19 (*SE* = 0.1), with a corresponding standard deviation of *τ* = 0.43, indicating an overall large level of heterogeneity among the included studies. The test revealed significant funnel plot asymmetry (*z* = 2.90, *p* = 0.0038), indicating potential publication bias in the included studies. However, the trim-and-fill method estimated the adjusted effect size as the standard error approached zero to be *b* = 0.17 (95% CI [−0.74, 1.09]), suggesting that although bias may be present, the overall effect estimate remains relatively stable but imprecise due to the wide confidence interval.

Subsequent moderator analyses were conducted to explore potential sources of the observed heterogeneity. Several moderator variables—task type, sample size, stimuli type, outcome measures, and percentage of male participants—emerged as important contributors to variability in effect sizes.

Task type significantly accounted for heterogeneity across studies (*Q_M_*(2) = 7.83, *R*^2^ = 36.39%, *p* = 0.02), with passive hearing tasks yielding significantly larger effect sizes (*β* = 1.41, *SE* = 0.52, *p* = 0.006). However, because only a single study employed a passive-hearing paradigm, this pattern may partially reflect the imbalance in the distribution of task types. Therefore, the magnitude of this effect should be interpreted with caution, and its underlying mechanisms warrant further investigation in future research.

Sample size was also a significant moderator (*Q_M_*(1) = 3.63, *R*^2^ = 17.69%, *p* = 0.05), indicating a marginally significant negative association between sample size and effect size—larger samples tended to report slightly smaller effects (*β* = −0.0054, *SE* = 0.0028, *p* = 0.05).

Stimuli type showed a significant effect as well (*Q_M_*(2) = 6.49, *R*^2^ = 30.46%, *p* = 0.04), with synthesized tones eliciting significantly larger effect sizes compared to real speech or tonal continua (*β* = 1.34, *SE* = 0.54, *p* = 0.01).

Outcome measures was a strong predictor of heterogeneity (*Q_M_*(5) = 18.82, *R*^2^ = 68.31%, *p* = 0.002), where studies using mean accuracy as the outcome variable reported significantly smaller effect sizes (*β* = −0.74, *SE* = 0.30, *p* = 0.01).

Although percentage of male participants did not significantly explain between-study heterogeneity (*Q*_M_(1) = 3.15, *R*^2^ = 25.06%, *p* = 0.07), there was a trend suggesting that higher proportions of male participants were associated with smaller effect sizes (*β* = −4.69, *SE* = 2.61, *p* = 0.07).

### 3.3 Effects of second language learning experience

**Figure 4:**
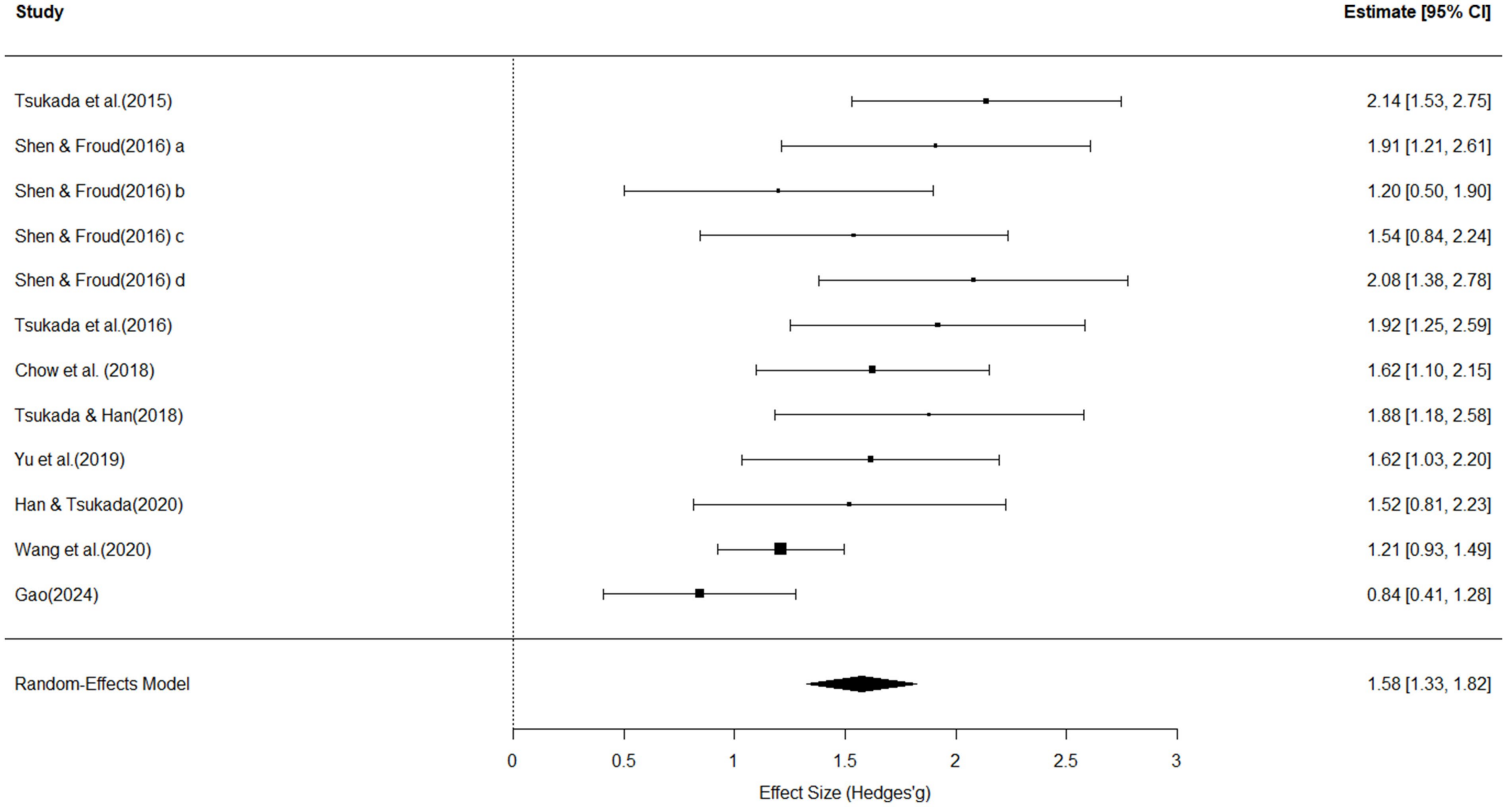
Forest plot for effects of L2 experience on perception, with individual and pooled effect sizes (Hedges’ *g*), standard errors and 95% confidence intervals (size of the rectangles represents study weight; horizontal line indicates 95% CI; diamond indicates pooled effect size; diamond width represents its CI).

Figure 4 presents the meta-analytic results based on 12 studies examining the influence of L2 experience on Mandarin lexical tone perception. The overall effect size was statistically significant and of large magnitude (effect size = 1.58, 95% *CI* [1.33, 1.82], *p* < 0.01; see Figure 4). The test for heterogeneity was non-significant (*Q*(11) = 24.61, *p* = 0.01), indicating substantial variation in effect sizes across studies. The *I*^2^ value was 54.92%, suggesting that over half of the variance in observed effect sizes reflects true between-study differences. The estimated between-study variance was *τ*^2^ = 0.09 (*SE* = 0.0054), corresponding to a standard deviation of τ = 0.31. Egger’s regression test revealed significant funnel plot asymmetry (*z* = 2.45, *p* = 0.01), indicating potential publication bias. The estimated effect size as the standard error approached zero was *b* = 0.62 (95% CI [−0.14, 1.39]).

Moderator analysis showed that sample size significantly accounted for heterogeneity across studies (*Q_M_*(1) = 3.54, *R*^2^ = 32.26%, *p* = 0.05), indicating larger samples tended to report slightly smaller effects (*β* = −0.004, *SE* = 0.0021, *p* = 0.05). Moreover, outcome measures was also a strong predictor of heterogeneity (*Q_M_*(3) = 8.52, *R*^2^ = 62.11%, *p* = 0.03), where studies using mean accuracy as the outcome variable reported significantly smaller effect sizes (*β* = −0.32, *SE* = 0.28, *p* = 0.01).

## 4 Discussion

The meta-analytic results revealed a statistically significant and robust effect of Mandarin tone perception across studies, with overall large effect sizes, confirming that tone perception differences are both reliable and theoretically meaningful. Notably, substantial heterogeneity was observed, and several methodological factors were found to significantly account for this variability. In particular, task type, stimuli type, outcome measure, sample size and the proportion of male participants emerged as key moderators explaining between-study differences.

Studies that employed passive hearing tasks, used synthesized stimuli, and adopted accuracy rate and A-prime score as the outcome measure consistently yielded larger effect sizes. These methodological features may enhance perceptual clarity and consistency, possibly due to more salient acoustic cues and reduced cognitive demands. Additionally, studies with a higher proportion of female participants tended to report larger effect sizes, suggesting that female listeners may be more attuned to subtle tonal differences, consistent with previous findings on gender-related perceptual sensitivity (Chen et al., 2024). Moreover, moderate sample sizes were sufficient to detect significant effects when examining the influence of native prosodic background. This suggests that increasing sample size is often confounded with participant homogeneity. Future research should therefore pay closer attention not only to sample size but also to the balance and diversity of participant characteristics. The studies included in the present review—and the accompanying meta-analytic patterns—provide a useful reference point for designing such future work.

Taken together, these findings highlight the importance of careful experimental design—including the selection of task format, stimulus type, and outcome metric—when investigating Mandarin lexical tone perception. Thoughtful control of these factors can improve the sensitivity and generalizability of results, particularly in studies targeting cross-linguistic tone perception.

Furthermore, these results cumulatively and consistently demonstrate that the three factors examined—tonal features of height and contour, native language prosodic background, and L2 experience—account for substantial variability in the effect sizes observed across studies. These patterns are made especially clear through the strengths of the meta-analytic approach, which allows systematic synthesis across heterogeneous findings. Taken together, the results indicate that these three factors play a critical role in shaping the perception of Mandarin tones. In the following sections, we examine each factor in greater detail.

### 4.1 Tone height and tone contour and their potential effects on tone perception

Identification studies consistently demonstrate a robust perceptual hierarchy among the four Mandarin tones: T1 ≈ T4 > T3 > T2, indicating that T1 and T4 are the most accurately identified, followed by T3, with T2 being the most difficult (Gao, 2024; So and Best, 2010; Li, 2016; Hao, 2018). This pattern is especially pronounced among listeners from non-tonal language backgrounds with no prior exposure to Mandarin, but it has also been observed in tone language speakers and even native Mandarin speakers. Importantly, manipulations of different experimental paradigms further show that this perceptual ranking is not limited to laboratory settings with isolated syllables; it also holds in real-word processing across different syllable positions, such as word-initial and word-final tones (Hao, 2018). Moreover, this perceptual hierarchy is further supported by tone discrimination studies. Across multiple investigations, the T2–T3 pair is consistently reported as the most difficult to discriminate, whereas pairs involving T1 or T4—such as T1–T2, T1–T3, T1–T4, T2–T4, and T3–T4—tend to yield the highest accuracy rates (So and Best, 2010; Tsukada et al., 2015; Tsukada et al., 2016; Tsukada and Han, 2018). These results collectively reinforce the observation that T1 and T4 are perceptually more robust, while T2 is the most confusable, regardless of listener background.

This trend aligns with earlier research findings across decades, all of which consistently show that T1 and T4 are the easiest to perceive, while T2 is the most difficult (Gandour, 1983; Wang et al., 1999). Moreover, this perceptual hierarchy aligns with findings from developmental research, which show that the order of tone acquisition in Mandarin-speaking children mirrors this perceptual trend (Li and Thompson, 1977; Tse, 1978). Although perhaps surprising at first glance, this alignment is intuitive: accurate perception is a necessary foundation for successful acquisition (Flemming, 2004). Thus, the convergence of behavioral, developmental, and cross-linguistic findings—especially those synthesized in the current meta-analysis—suggests that the observed tone perception hierarchy is not merely task- or context-dependent, but rather reflects deeper acoustic-perceptual properties intrinsic to the Mandarin tonal system.

These intrinsic properties are closely linked to the acoustic dimensions of tone height and tone contour, as discussed in Section 1.1. T1 is a high-level tone, whereas T2, T3, and T4 are contour tones. Interestingly, the numerical imbalance (1 level tone vs. 3 contour tones) does not translate into the perceptual disadvantage of T1. In fact, T1 is the most accurately perceived tone. This advantage appears primarily tied to its high pitch register. Several studies have suggested that high tones are generally more perceptually salient than low tones, possibly because higher pitch is acoustically more prominent and attention-grabbing (Francis et al., 2008; Wong and Diehl, 2003). This hypothesis was directly supported by Francis et al. (2008), who trained native speakers of Mandarin and English to perceive six Cantonese tones—three in the high register and three in the low register. Regardless of the contour shape, participants from both language backgrounds performed significantly better on the high-register tones. These findings suggest that tone height—particularly high pitch—is a fundamental factor influencing perceptual salience.

On the other hand, tone contour becomes more relevant when comparing among contour tones. T2 (35), T3 (214), and T4 (51) differ in pitch trajectory complexity: T3 is often considered the most complex due to its falling–rising contour, followed by T4 (simple fall), and then T2 (simple rise). However, empirical data consistently show that T2 is harder to perceive than T3, indicating that contour complexity alone does not determine perceptual difficulty. An alternative explanation comes from Flemming’s (2004) “Maximize Distinctiveness” theory, which proposes that contrasts occupying greater acoustic space are more perceptually distinctive. By this account, T4 spans the largest pitch range (from 5 to 1), T3 spans 3 units (1 to 4), and T2 covers the smallest range (3 to 5), making it the least distinct. This distribution is supported by Xu (1997), who provided a detailed analysis of tonal pitch trajectories in Mandarin. Hence, in contour tones, it is the pitch range occupied by the tone, rather than the complexity of pitch direction, that better predicts perceptual salience.

However, this pitch-range-based advantage appears to apply primarily to contour tones. T1 (55), which spans the narrowest pitch range among all Mandarin tones, is nevertheless the easiest to perceive. This suggests that when tone height and contour interact, pitch height may exert a dominant influence on perceptual salience. This is specifically manifested in the fact that, within the high-pitch register, level tones such as T1 (55) are perceptually more accurately than contour T2 (35). This perceptual pattern may be rooted in historical tone evolution and early language acquisition. Historically, Chinese tones likely evolved from primarily level pitch patterns (Shen, 2020). For instance, consider a syllable like [a^1^] and the other [ba^1^], both of which originally carried T1 (indicated by the numeral ‘1’). Due to the articulatory nature of the voiced stop [b], [ba^1^] would have had a slightly lower pitch onset, resulting in a naturally rising pitch contour. After [b] was lost through phonological erosion, the rising pitch pattern was retained, and perhaps to some extend exaggerated, to preserve the contrast between the two syllables. This led [ba^1^] to evolve into [a^2^] (superscript numerals ^1^ and ^2^ denote T1 and T2), with the tonal contrast alone distinguishing the two syllables. Thus, what was initially a coarticulatory effect associated with voiced consonants became phonologized as a new tone (T2), while [a^1^] retained the original T1. From this diachronic perspective, level tones formed the baseline, with contour tones emerging later and requiring finer perceptual resolution. Developmental data further corroborate this view. Research consistently shows that high-level tones (like T1) are acquired earliest by both Mandarin and Cantonese-speaking children (Li and Thompson, 1977), reinforcing the idea that high, level tones hold a foundational role in tone perception and acquisition across different tone systems.

Although the effects of tone height and tonal span on tone perception have been relatively well established through the above analyses, one unresolved issue concerns the role of pitch direction—specifically, whether rising or falling contours have a perceptual advantage over the other. This question remains open due to the asymmetry in Mandarin’s tone inventory, which lacks directly comparable pairs like ‘15’ and ‘51’ that would allow for controlled comparisons of directional effects. Future research should explore this issue using tone systems in other languages where directional contrast is more symmetrically represented and minimally confounded by other factors. Nonetheless, such systems are rare and might pose additional experimental challenges.

### 4.2 Native language prosodic background and its influence on tone perception

Our meta-analytic results reveal that speakers of non-tonal and non-pitch-accent languages consistently perform worse in tone identification and discrimination tasks than speakers of tone languages and pitch-accent languages. However, this overall disadvantage is not absolute; rather, it is shaped by the specific characteristics of listeners’ native prosodic systems.

For speakers of non-tonal and non-pitch-accent languages, their perception tends to rely primarily on the inherent perceptual salience of individual Mandarin tones, as discussed in Section 4.1. For instance, English speakers are reported to perceive T1 and T4 relatively effortlessly but tend to confuse the two (So and Best, 2010), suggesting that while they can detect the most perceptually salient tones T1 and T4 in the system, they struggle to resolve finer differences in pitch contour between the two. Similarly, Korean listeners can distinguish highly salient tones such as T1 and T4 from the others but have difficulty with the more acoustically subtle T2–T3 contrast (Han and Tsukada, 2020). Moreover, Danish and Indonesian speakers show high accuracy in perceiving T4 but significant difficulty with T2 (Chow et al., 2018; Gao, 2024). Collectively, these findings suggest that non-tonal, non-pitch-accent listeners are attuned to the most acoustically prominent tones (T1 and T4), which are distinguished by high pitch values and large pitch span. However, they lack sufficient sensitivity to the more nuanced pitch trajectories of T2 and T3, leading to frequent perceptual dilemma.

By contrast, pitch-accent language speakers demonstrate significantly better tone perception than non-tonal speakers and are more adept at perceiving finer contour distinctions. For example, both Japanese and Swedish pitch-accent listeners outperformed English and Danish speakers in Mandarin tone tasks (So and Best, 2010; Gao, 2024). This advantage is often attributed to their experience with pitch-based contrasts in their native languages. For instance, So and Best (2010) hypothesized that Japanese listeners would benefit from positive transfer when perceiving T4 (51) in one syllable, due to the similarity between this contour and the HL pitch patterns found in Japanese two-syllable words. However, their findings did not support this prediction, suggesting that the observed advantage among pitch-accent listeners is not simply due to cross-linguistic similarity in mere pitch patterns. Instead, the advantage may stem from their enhanced ability to process pitch variation at a finer resolution, cultivated through regular use of pitch cues in their L1 prosody. Interestingly, because they are not constrained by a pre-existing tonal category system, pitch-accent speakers often outperform even tone language speakers when perceiving unfamiliar tones.

Tone language speakers, not surprisingly, exhibit the highest overall performance in tone perception tasks. They are better able to detect fine-grained pitch differences within the same register, such as distinguishing between Mandarin T1 (55) and T2 (35), which differ only subtly in pitch trajectory. However, tone language listeners are also more susceptible to the influence of their native tone inventory. According to the Perceptual Assimilation Model (PAM), listeners tend to assimilate non-native tone stimuli to the closest tonal categories in their L1. This assimilation can lead to both facilitation and interference. For example, Cantonese has both 55 and 53 tones, which may help Cantonese speakers distinguish between Mandarin T1 (55) and T4 (51) better than other groups (So and Best, 2010). Similarly, Thai speakers—whose tonal inventory closely resembles Mandarin T1, T2, and T4—perform at near-native levels in Mandarin tone perception (Krishnan et al., 2010). However, in many cases, assimilation leads to perceptual interference. This typically occurs when two tones from the target language are both mapped onto a single L1 category. For example, both Mandarin T2 (35) and T3 (214) may be assimilated to Cantonese tone ‘25’, causing difficulty in differentiating the two. As a result, Cantonese speakers often perform worse on T2–T3 discrimination than English speakers (So and Best, 2010). A similar pattern has been observed among Hmong speakers, who struggle to distinguish Mandarin T1 (55) and T2 (53) because both are perceived as variants of a single high tone in Hmong (Wang, 2013).

Taken together, the influence of native prosodic background on tone perception is not a matter of absolute advantage or disadvantage across language types, but rather depends on the specific way in which the L1 prosodic system interacts with the target tone system. Non-tonal and non-pitch-accent speakers generally operate with lower perceptual resolution for pitch contours compared to tonal and pitch accent listeners. This disparity is likely linked to hemispheric differences in auditory processing. Neuroimaging studies have shown that non-tonal language speakers primarily engage the right temporal lobe when processing pitch variation, whereas tone and pitch-accent language speakers exhibit greater activation in left-hemisphere regions associated with linguistic processing (Gandour et al., 2000; Zatorre and Belin, 2001). This left-lateralized processing is thought to facilitate more categorical and fine-grained perception of pitch patterns in tonal and pitch-accent language users. On the one hand, such categorization—driven by perceptual assimilation—can be beneficial for tone language speakers. It allows them to efficiently map unfamiliar L2 tones onto the most similar L1 tone categories, thus enabling rapid and accurate perception. On the other hand, this same process may also lead to interference when the tonal categories of the target language do not align neatly with those of the listener’s native language. In these cases, multiple L2 tones may be assimilated to a single L1 category, resulting in confusion and reduced perceptual accuracy. As a result, tone language speakers may occasionally perform worse than non-tonal listeners, especially when the L1 tonal system introduces misleading equivalences.

### 4.3 L2 experience and its role in perception of Mandarin tones

Meta-analytic results indicate a generally positive relationship between second language (L2) experience and Mandarin tone perception. As learners accumulate more years of study, their overall tone perception improves—not only for naturally produced tones but also for synthesized tone continua (Shen and Froud, 2016). In the early stages of learning, L2 learners tend to rely on perceptually salient cues and are most successful at identifying tones with strong acoustic distinctiveness, particularly T1 and T4. With increased exposure and training, learners begin to develop finer perceptual sensitivity, gradually acquiring the ability to distinguish tones with more subtle pitch contour differences, such as T2 (35) from T4 (51) or T2 (35) from T1 (55) (Tsukada et al., 2015). This shift suggests that improvement in tone perception involves more detailed tracking of pitch movements over time, especially within the high-pitch register.

However, this positive correlation between L2 experience and perceptual accuracy is not strictly linear. Wang et al. (2020), for example, examined four groups of English-speaking learners of Mandarin: naïve, beginner (1 month), intermediate (1 year), and advanced (2 years). The study found no significant improvement in tone perception among the beginner and intermediate groups compared to naïve listeners. Only the advanced group exhibited performance levels approaching that of native speakers. Similarly, Shen and Froud (2016) reported that only learners with at least three semesters of formal Mandarin study reached perceptual accuracy comparable to native speakers. These findings suggest that Mandarin tone perception poses a persistent challenge for L2 learners, especially learners from non-tonal background, and may require at least 1.5 years of sustained learning for substantial improvement to emerge.

Moreover, both the acoustic characteristics of specific tones and the prosodic background of the learner’s native language significantly mediate the effects of L2 experience, as discussed in Sections 4.1 and 4.2. Consistent across studies and language backgrounds, L2 learners—regardless of experience level—consistently perceive T1 and T4 more accurately than other tones, reinforcing the notion that these tones possess universal perceptual advantages. In contrast, the T2–T3 pair continues to present the greatest difficulty. Nevertheless, studies have shown that learners’ ability to discriminate this pair improves significantly over time. For instance, Tsukada et al. (2016) and Tsukada and Han (2018) found that both Japanese-speaking and Korean-speaking L2 learners of Mandarin demonstrated improved perception of T2–T3 compared to naïve listeners, even among learners at the beginner level.

The influence of L1 prosodic background is also evident. Learners from pitch-accent language backgrounds, such as Japanese, generally outperform learners from non-tonal language backgrounds, such as English, especially in distinguishing acoustically similar tones like T2 and T3 (Tsukada, 2022). Despite both groups being advanced learners, Japanese speakers show enhanced sensitivity to fine-grained pitch contours, likely due to their experience with pitch-based lexical contrasts in their native language.

In contrast, tone language speakers exhibit mixed outcomes. On the one hand, learners from tonal backgrounds such as Thai tend to outperform those from non-tonal backgrounds like Indonesian (Chow et al., 2018), possibly due to shared pitch-related processing mechanisms. On the other hand, tonal L1s can interfere with the perception of non-native tones, especially when L1 and L2 tones are acoustically similar but functionally distinct. This interference is particularly evident in Cantonese-speaking learners of Mandarin. Due to overlapping pitch patterns in the two tone systems of Mandarin and Cantonese, Cantonese Mandarin learners often struggle to distinguish Mandarin tones such as T2 and T3. As reported by Tsukada et al. (2015), these learners performed worse than English-speaking learners with similar L2 proficiency levels, highlighting the potential for negative transfer when L1 tonal categories conflict with L2 tone contrast.

## 5 Conclusion

This meta-analysis synthesized data from 18 empirical studies on Mandarin tone perception and revealed overall large effect sizes, indicating that perceptual variation across tonal features of height and contour, listeners’ prosodic backgrounds, and L2 experience is both statistically robust and theoretically meaningful. Importantly, substantial heterogeneity in effect sizes was explained by several key methodological moderators, including task type, stimulus type, outcome measure, sample size, and the proportion of male participants. Specifically, studies that employed passive hearing tasks, used synthesized stimuli, and measured performance through accuracy rate consistently yielded larger effect sizes, suggesting that these features enhance the perceptual salience of tone contrasts while reducing cognitive processing demands. Moreover, studies with a greater proportion of female participants tended to show stronger effects, potentially reflecting gender-related differences in prosodic sensitivity. Notably, larger sample sizes did not consistently correspond to stronger effect sizes. This pattern suggests that increases in sample size alone do not necessarily lead to stronger or more reliable effects, as sample size often interacts with—and may even be constrained by—participant homogeneity, which can in turn attenuate the observed outcomes. Future research should therefore attend not only to the absolute number of participants but also to the balance and diversity of participant characteristics when designing tone-perception studies.

Building on the current meta-analysis, three central and interrelated questions in the field of lexical tone perception were examined in depth:

Tone Type Effects based on Tone Height and Tone Contour: Tone 1 and Tone 4 were consistently perceived with the highest accuracy across all listener groups, while Tone 2 and Tone 3 remained most difficult. This pattern reflects the influence of tone height and tone contour, with higher pitch levels and contours spanning larger pitch ranges generally yielding greater perceptual salience.

Native Prosodic Background: Listeners’ L1 prosodic systems significantly influenced perception. Non-tonal speakers relied more on general acoustic cues, while pitch-accent and tone language speakers showed more refined pitch processing. However, tone language speakers were also vulnerable to perceptual assimilation, which could both aid and hinder performance depending on L1-L2 tone similarity.

L2 Experience: Greater L2 experience improved tone perception, but progress was tone-specific and nonlinear. Learners typically improved on T1 and T4 earlier, while T2 and T3 remained difficult even at advanced levels. Moreover, L1 background continued to shape perception throughout the learning process, and perceptual assimilation effects sometimes led tonal L1 learners to perform worse than non-tonal learners.

These findings highlight that L2 tone acquisition cannot be adequately explained by exposure duration alone. Instead, it reflects a dynamic interaction among perceptual salience, L2-to-L1 assimilation mechanisms, and individual learner factors. Overall, native prosodic background plays a more prominent role during the early stages of learning, while intrinsic tone properties—such as pitch height and contour complexity—become more determinative as learners advance.

In sum, this meta-analysis provides compelling evidence that Mandarin tone perception is shaped by multiple interacting dimensions—namely, the acoustic-perceptual properties of the tones themselves, the prosodic experience of the listener, and the extent of L2 exposure. Theoretically, our findings underscore the importance of jointly considering tonal features and listeners’ native prosodic background in research on tone perception. Models of L2 tone acquisition are suggested to reflect the asymmetrical effects of different tonal features as well as the facilitative and interfering influences of L1 prosodic systems. These results further highlight the need for tone perception models to incorporate perceptual salience hierarchies and assimilation dynamics as core components. Pedagogically, the findings point to several practical implications. Tone training for L2 Mandarin learners should place early and explicit emphasis on contour tone distinctions, particularly the notoriously confusable T2 and T3. The ability to accurately distinguish between these two tones may serve as a diagnostic indicator of learners’ overall tone perception development. Furthermore, instructional materials and teaching strategies should be tailored to learners’ L1 prosodic backgrounds, taking into account common patterns of cross-linguistic assimilation and potential areas of interference.

Finally, future research would benefit from longitudinal and neurocognitive approaches that trace how tone perception develops over time and how it is supported by dynamic shifts in cortical processing. Additional perceptual factors—such as noise conditions, music training, and voice-related characteristics (e.g., the well-established influence of voice quality on the perception of Mandarin Tone 3)—also warrant further investigation. More meta-analytic work is also needed across other tone languages to determine whether the patterns observed here generalize beyond Mandarin and to identify potential typological universals in tone perception.
